# Exogenous spermidine (Spd) improves the tolerance to alkaline-salt stress in potato

**DOI:** 10.1186/s12870-026-09656-7

**Published:** 2026-08-04

**Authors:** Yuying Fu, Qianqian Wang, Yuwei Ge, Huajun Liao, Chongchong Yan

**Affiliations:** 1 Institute of Vegetables, Anhui Academy of Agricultural Sciences, Hefei, Anhui 230031 China; 2 Key Laboratory of Horticultural Crop Germplasm Innovation and Utilization (Co-construction by Ministry and Province), Hefei, Anhui 230031 China; 3 Anhui Provincial Key Laboratory for Germplasm Resources Creation and High-Efficiency Cultivation of Horticultural Crops, Hefei, Anhui 230031 China

**Keywords:** Potato, Alkaline-salt stress, Spermidine, Antioxidant system, Transcriptome, WGCNA

## Abstract

**Background:**

Potato (*Solanum tuberosum* L.) is the world’s fourth most important staple crop, but its production is increasingly threatened by soil salinization, particularly alkaline-salt stress caused by excessive NaHCO_3_. Although exogenous spermidine (Spd) has been reported to alleviate abiotic stresses in various plants, its physiological and molecular mechanisms in conferring alkaline-salt tolerance in potato remain largely unknown.

**Results:**

The results demonstrated that exogenous Spd enhances alkaline-salt tolerance in potato by increasing antioxidant enzyme activities and maintaining osmotic balance. RNA sequencing (RNA-seq) and weighted gene co-expression network analysis (WGCNA) revealed obvious tissue-specific transcriptional reprogramming in potato under Spd-combined alkaline-salt stress, with 5,968 differentially expressed genes (DEGs) identified in leaves and only 187 in roots. KEGG pathway analysis indicated that Spd mainly regulates plant hormone signal transduction, carbon metabolism and photosynthesis pathways in leaves, as well as ribosome and energy metabolism-related pathways in roots. Three candidate hub genes, *StHK4*, *StbZIP27* and *StERF106*, were screened from key Spd-responsive modules. Among them, *StHK4* exhibited the highest upregulation ( Log_2_FC = 3.40) and positively mediates the cytokinin-ABA signaling pathway, *StbZIP27* functions in bZIP-dependent regulatory pathways to promote osmotic adjustment and antioxidant defense, and *StERF106* integrates ethylene signaling and reactive oxygen species (ROS) scavenging pathways. These pathways jointly improve membrane stability, maintain osmotic balance and enhance ROS-scavenging capacity, ultimately improving potato tolerance to alkaline-salt stress.

**Conclusions:**

This study demonstrates that exogenous Spd enhances alkaline-salt tolerance in potato by modulating multiple physiological processes and transcriptional networks. The screened candidate hub genes (*StHK4*, *StbZIP27*, and *StERF106*) serve as potential pivotal regulators responsible for Spd-induced stress tolerance, providing valuable gene resources and novel mechanistic insights for understanding polyamine-mediated stress adaptation in potato.

**Supplementary Information:**

The online version contains supplementary material available at https://doi.org/10.1186/s12870-026-09656-7.

## Background

Soil salinization is a major global stressor that severely limits plant growth and agricultural productivity. Alkaline-salt stress, characterized by elevated pH and high Na^+^ concentrations, reduces the solubility of rhizosphere trace elements, impairs root water and nutrient uptake, and causes direct tissue damage and ion toxicity [1, 2]. Such stress triggers osmotic imbalance and cellular dehydration, inducing the accumulation of osmolytes such as proline to maintain cellular water potential [3, 4]. Concurrently, excessive ROS accumulate particularly H_2_O_2_, causing lipid peroxidation and DNA damage [5]. To counteract this, plants activate an antioxidant defense system comprising superoxide dismutase (SOD), peroxidase (POD), catalase (CAT), and ascorbate peroxidase (APX), with salt-tolerant species often exhibiting enhanced antioxidant capacity [6, 7].

Potato (*Solanum tuberosum* L.), the world’s fourth most important staple crop, supports approximately 1.3 billion people and holds immense nutritional value [8, 9]. However, potato is moderately sensitive to salt and alkaline-salt environments [10], and soil salinization increasingly threatens its production. Yield losses begin when soil pH exceeds 7.0, and severe alkalinity can inhibit emergence, increase common scab incidence, and reduce tuber quality. Alkaline-salt stress directly causes leaf chlorosis, wilting, and chloroplast damage, partly because elevated abscisic acid (ABA) triggers stomatal closure and suppresses photosynthesis [11, 12]. Multiple mechanisms contribute to these losses: stress alters chlorophyll and soluble sugar levels, disrupts starch biosynthesis [13], and upregulates the flowering suppressor *StBBX24*, delaying the vegetative-to-reproductive transition [14]. Physiologically, stress elevates malondialdehyde (MDA), promotes osmolyte accumulation, activates antioxidant defenses, and upregulates phytohormone signaling genes (including polyamines), ultimately impairing tuber yield and quality [11].

Polyamines, mainly putrescine (Put), spermidine (Spd), and spermine (Spm), are essential regulators of plant growth and stress tolerance [15]. Exogenous Spd application has been verified to enhance saline-alkaline tolerance in multiple crop species, including wheat (*Triticum aestivum* L.), tomato (*Solanum lycopersicum* L.), rice (*Oryza sativa* L.), rapeseed (*Brassica napus* L.), oat (*Avena sativa* L.), and grapevine (*Vitis vinifera* L.), by enhancing antioxidant enzyme activity, regulating stress-related gene expression, promoting endogenous polyamine accumulation, and alleviating oxidative damage [16–21]. Despite this progress, the physiological and molecular mechanisms through which Spd confers alkaline-salt stress tolerance in potato remain unclear.

The integration of transcriptome sequencing with WGCNA has become a powerful approach to dissect plant stress responses at the systems level, enabling the identification of key regulatory genes and pathways modulated by exogenous substances [22]. In potato, transcriptomic approaches have already identified pivotal transcription factors such as *StbZIP1* that positively regulate saline-alkali tolerance [23]. In this study, we used the potato cultivar ‘Huishu No.5’ to systematically investigate how foliar-applied Spd (1 mmol·L^− 1^) alleviates alkaline-salt stress (300 mmol·L^− 1^ NaHCO_3_) at the physiological, biochemical, and transcriptomic levels. Our results provide new mechanistic insights into Spd-mediated alkaline salt tolerance and offer theoretical guidance and gene resources for saline-alkali-tolerant potato cultivation and breeding.

## Methods

### Plant material and treatments

The potato (*Solanum tuberosum* L.) cultivar ‘Huishu No. 5’ used in this study was bred and is maintained by the Potato Breeding Research Group, Vegetable Research Institute, Anhui Academy of Agricultural Sciences (Hefei, China). This widely cultivated fresh-eating potato cultivar in Anhui Province possessed a stable genetic background and mature tissue culture system, which guarantees reliable experimental reproducibility. All plant materials were sourced from the institute’s germplasm repository, and no wild plant materials were collected in this experiment.

Based on a published potato stress protocol [24], 300 mmol·L^− 1^ NaHCO_3_ was preliminarily adopted as the alkaline-salt stress concentration. A gradient screening test with 100–400 mmol·L^− 1^ NaHCO_3_ further verified that this concentration induced typical stress symptoms without seedling death, which was suitable for assessing Spd-mediated stress tolerance. Meanwhile, 1 mmol·L^− 1^ Spd was set according to a validated tomato treatment method [25]. Preliminary tests confirmed that the established treatment regime caused obvious growth inhibition and leaf chlorosis, while exogenous Spd effectively relieved stress damage with no phytotoxicity, validating the reliability of this system for subsequent physiological and transcriptomic analyses.

The experiment was conducted from April to November 2024 in the tissue culture laboratory and greenhouse of the Anhui Academy of Agricultural Sciences. Virus-free plantlets of cultivar ‘Huishu No. 5’ were propagated through leaf tip culture and multiplication. Uniform, virus-free potato plantlets were selected, and their roots were sterilized with 0.1% HgCl_2_ for 5 min, followed by thorough rinsing with distilled water. The plantlets were then uniformly transplanted into hard plastic trays (50 plants per tray; tray dimensions: 20 cm height, 40 cm length, 25 cm width) containing vermiculite, with drainage holes at the bottom. They were cultivated in an artificial climate chamber set at 25 ℃/20 ℃ (day/night ± 1 ℃), with a 14/10 h light/dark photoperiod and 60%–70% relative humidity.

When the third true leaf had fully expanded, uniformly grown seedlings were selected and transferred from the artificial climate chamber to pots in the greenhouse for natural cultivation. After two weeks of natural cultivation, one seedling was retained per pot. At the budding stage, the seedlings were randomly divided into four treatment groups: CK (Control), distilled water; S (Spd treatment), 1 mmol·L^− 1^ Spd; TS (alkaline-salt stress + Spd treatment), 300 mmol·L^− 1^ NaHCO_3_ + 1 mmol·L^− 1^ Spd; T (alkaline-salt stress), 300 mmol·L^− 1^ NaHCO_3_+ distilled water.

Spd solution was applied as a foliar spray daily at 16:00 until runoff, for a total of 3 consecutive days. Each treatment consisted of 20 seedlings with three replicates. NaHCO_3_ treatment was initiated after the third Spd application. The NaHCO_3_ solution was applied by evenly irrigating each pot with 1 L of 300 mmol·L^− 1^ NaHCO_3_ solution. At 24 h after NaHCO_3_ treatment, the 2nd to 3rd true leaves below the leaf apex and the taproot were collected for physiological measurements. Tubers were harvested on 15 November 2024; ten pots per treatment were randomly sampled to calculate mean fresh tuber yield per pot.

### Measurements of physiological parameters

Frozen leaf or root tissue (0.1 g) was homogenized following the manufacturer protocols of the corresponding commercial assay kits (Yeasen Life Science, Shanghai, China; Solarbio, Beijing, China). All physiological indices were quantified strictly in accordance with kit instructions, with three biological replicates and three technical replicates set for each sample.

MDA content was measured by the thiobarbituric acid (TBA) method: tissue samples were homogenized at a ratio of 1:9 (w/v) to prepare a 10% homogenate, and centrifuged at 3,500–4,000 rpm for 10 min. The supernatant (0.1 mL) was mixed with 0.1 mL of reagent 1, 3.0 mL of reagent 2, and 1.0 mL of reagent 3. For the control tube, 1.0 mL of 50% glacial acetic acid was used instead of reagent 3. The mixture was heated in a boiling water bath at 95 ℃ for 40 min, cooled, and centrifuged at 3,500–4,000 rpm for 10 min. The absorbance was read at 532 nm and corrected at 600 nm using a 1 cm cuvette. MDA content was calculated using a standard curve prepared with the provided standard (10 nmol·mL^− 1^) and expressed as nmol per mg protein.

SOD activity was determined using the NBT photochemical reduction method: tissue samples were homogenized in pre-cooled PBS, centrifuged at 4 ℃, and the supernatant was collected. The reaction mixture contained 20 µL of supernatant, 160 µL of NBT/enzyme working solution, and 20 µL of reaction initiator. After incubation at 37 ℃ for 30 min, the absorbance was measured at 560 nm. One unit of SOD activity was defined as the amount of enzyme required to inhibit 50% of NBT photoreduction under the assay conditions.

POD activity was measured by the guaiacol oxidation method: tissue samples were homogenized in extraction buffer at a ratio of approximately 1:10 (w/v), and centrifuged at 8,000 × g for 10 min at 4 ℃. The supernatant was collected and kept on ice. The reaction mixture contained 15 µL of supernatant, 270 µL of distilled water, 520 µL of reagent 1, 130 µL of reagent 2 working solution, and 135 µL of reagent 3. Absorbance at 470 nm was recorded at 30 s and 1 min 30 s after reaction initiation using a 1 mL glass cuvette. One unit of POD activity was defined as the amount of enzyme causing a 0.01 increase in absorbance per minute per gram of tissue.

CAT activity was assayed using the ammonium molybdate colorimetric method at 405 nm: tissue samples were homogenized in physiological saline (1:9, w/v) and centrifuged at 2,500 rpm for 10 min. Reagents 1 and 2 were pre-warmed at 37 ℃ for 10–20 min before use. The reaction mixture contained 1.0 mL of reagent 1, 0.1 mL of reagent 2, and 0.05 mL of supernatant. After incubation at 37℃ for exactly 1 min, 1.0 mL of reagent 3 and 0.1 mL of reagent 4 were added to terminate the reaction, and the absorbance was recorded at 405 nm using a 0.5 cm light-path cuvette. One unit of CAT activity was defined as the amount of enzyme catalyzing the decomposition of 1 µmol of H_2_O_2_ per second per mg of protein under the assay conditions.

APX activity was determined by monitoring the decrease in absorbance at 290 nm due to ascorbate oxidation: tissue samples were homogenized in extraction buffer (reagent 1) at a ratio of 1:9 (w/v), and centrifuged at 10,000 rpm for 10 min. The reaction mixture contained 100 µL of supernatant, 700 µL of reagent 1, 100 µL of reagent 2 working solution, and 100 µL of reagent 3. Absorbance at 290 nm was recorded at 10 s and 130 s after reaction initiation using a 1 cm quartz cuvette. The enzyme activity was calculated using the molar extinction coefficient of ascorbate (ε = 2.8 µmol^− 1^·mL^− 1^·cm^− 1^). One unit of APX activity was defined as the amount of enzyme oxidizing 1 µmol of ascorbate per minute per mg of protein.

Proline content was determined using the acidic ninhydrin colorimetric method: tissue samples were homogenized in 1 mL of extraction buffer (glacial acetic acid), incubated in a boiling water bath for 10 min with oscillation, and centrifuged at 10,000 × g for 10 min at room temperature. The supernatant (0.5 mL) was reacted with 0.5 mL of glacial acetic acid and 0.5 mL of acidic ninhydrin reagent in a boiling water bath for 30 min. After cooling, the absorbance was measured at 520 nm. Proline content was calculated from a standard curve and expressed as µg per g fresh weight.

Soluble sugar content was determined using the anthrone-sulfuric acid colorimetric method: tissue samples (approximately 0.1 g) were homogenized in 1 mL of distilled water, incubated in a boiling water bath for 10 min, and centrifuged at 8,000 × g for 10 min at room temperature. The supernatant was adjusted to a final volume of 10 mL with distilled water. An aliquot (200 µL) was mixed with 100 µL of working solution and 1000 µL of concentrated sulfuric acid, then incubated at 95 ℃ for 10 min. After cooling, the absorbance was measured at 620 nm. Soluble sugar content was calculated from a glucose standard curve and expressed as mg per g fresh weight.

### RNA sequencing and differentially expressed gene analysis

Potato leaf and root samples were sent to Biomarker Technologies (Beijing, China) for transcriptome sequencing. Total RNA was extracted using the CTAB method. RNA purity and concentration were assessed with a NanoDrop 2000 spectrophotometer (Thermo Scientific, Wilmington, DE, USA), and RNA integrity was evaluated using an Agilent 2100 Bioanalyzer system (Agilent Technologies, Santa Clara, CA, USA). After quality control, mRNA was enriched using oligo (dT)-coupled magnetic beads and then fragmented. First-strand and second-strand cDNA were synthesized, followed by end repair, A-tailing, adapter ligation, and size selection with AMPure XP beads. The cDNA library was quantified with a Qubit 3.0 fluorometer and validated using a Qsep400 high-throughput analysis system. Effective library concentration was determined by qPCR (library concentration > 2 nM). Sequencing was performed on Illumina NovaSeq 6000 platform.

Clean reads were mapped to potato reference genome DM v6.1 (*Solanum tuberosum*) using HISAT2 software. Differential expression analysis was performed using DESeq2 (fold change ≥ 2, false discovery rate < 0.05). Functional annotation of differentially expressed genes was carried out by aligning sequences against NR, Swiss-Prot, GO, COG, KOG, Pfam and KEGG databases using BLAST and HMMER software. KEGG pathway enrichment was analyzed with KOBAS 2.0, and significantly enriched pathways were identified at *P* < 0.05.

### Real-time quantitative PCR

Total RNA was extracted from each sample, and 500 ng of RNA was reverse-transcribed using the Hifair Ⅱ 1st Strand cDNA Synthesis SuperMix for qPCR (gDNA digester plus) (Yeasen, China). Real-time PCR was performed on a LightCycler 480 Ⅱ Real-Time PCR System (Roche, Switzerland). Primer sets were designed with Primer5 software, and the potato actin gene was used as an internal control (Table S2). The 20 µL reaction mixture contained 1 µL of diluted cDNA, 0.6 µL of each forward and reverse primer, 7.8 µL of ddH_2_O, and 10 µL of Hieff UNICON^®^ qPCR SYBR Green Master Mix (Yeasen, China). Relative transcript levels were calculated using the 2^−ΔΔCt method. Three biological replicates were used, and each reaction was performed in triplicate. All data are expressed as mean ± SD after normalization.

### WGCNA and candidate gene screening

A weighted gene co-expression network was constructed using the gene expression matrix, and phenotypic traits (alkaline-salt stress treatment, Spd treatment, proline content) were correlated to identify core modules. The core module genes were intersected with the DEGs from the TS vs. T comparison group to obtain key candidate genes. A weighted gene co-expression network was constructed based on normalized gene expression profiles; the soft threshold power was set to 12 to generate a scale-free network. Modules with eigengene-trait correlation coefficients > 0.7 were retained for subsequent correlation analysis.

### Statistical analysis

All experimental data were subjected to one-way analysis of variance (ANOVA) using IBM SPSS Statistics 21.0. Significant differences among treatments were determined by the Least Significant Difference (LSD) multiple comparison tests at α = 0.05.

## Results

### Effects of exogenous Spd on growth phenotype and tuber yield of potato under alkaline-salt stress

**Fig. 1:**
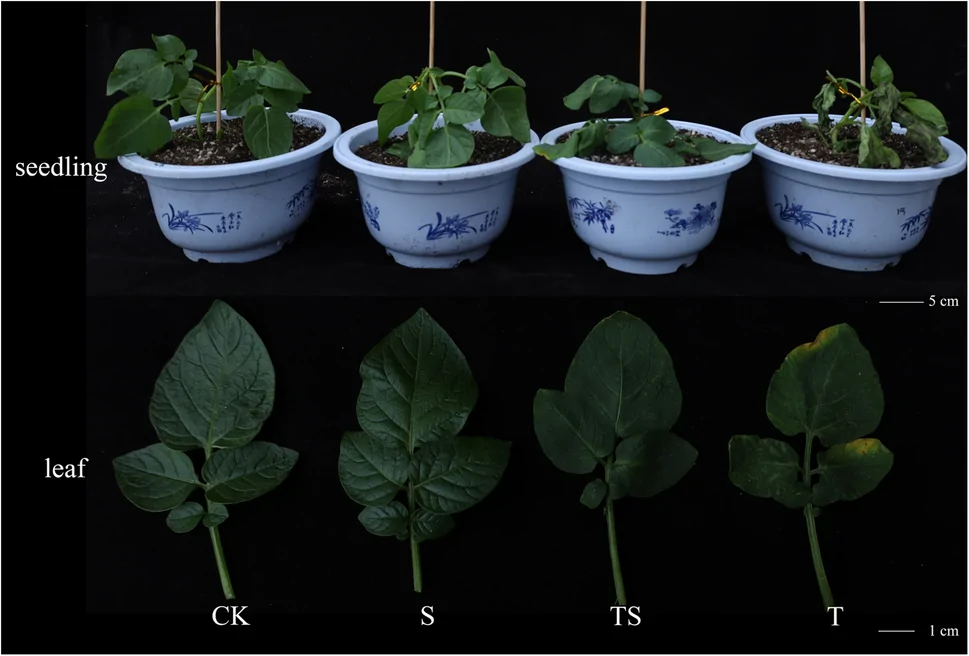
Morphological phenotypes of potato whole seedlings and detached functional leaves collected at the sampling stage. Upper panel: intact potted plants; lower panel: representative fully expanded leaves. Scale bars: 5 cm (whole plants), 1 cm (detached leaves). CK, distilled water control; S, foliar application of 1 mmol·L^− 1^ Spd under normal conditions; TS, combined treatment of 300 mmol·L^− 1^ NaHCO_3_ and 1 mmol·L^− 1^ Spd; T, single alkaline-salt stress of 300 mmol·L^− 1^ NaHCO_3_

**Fig. 2:**
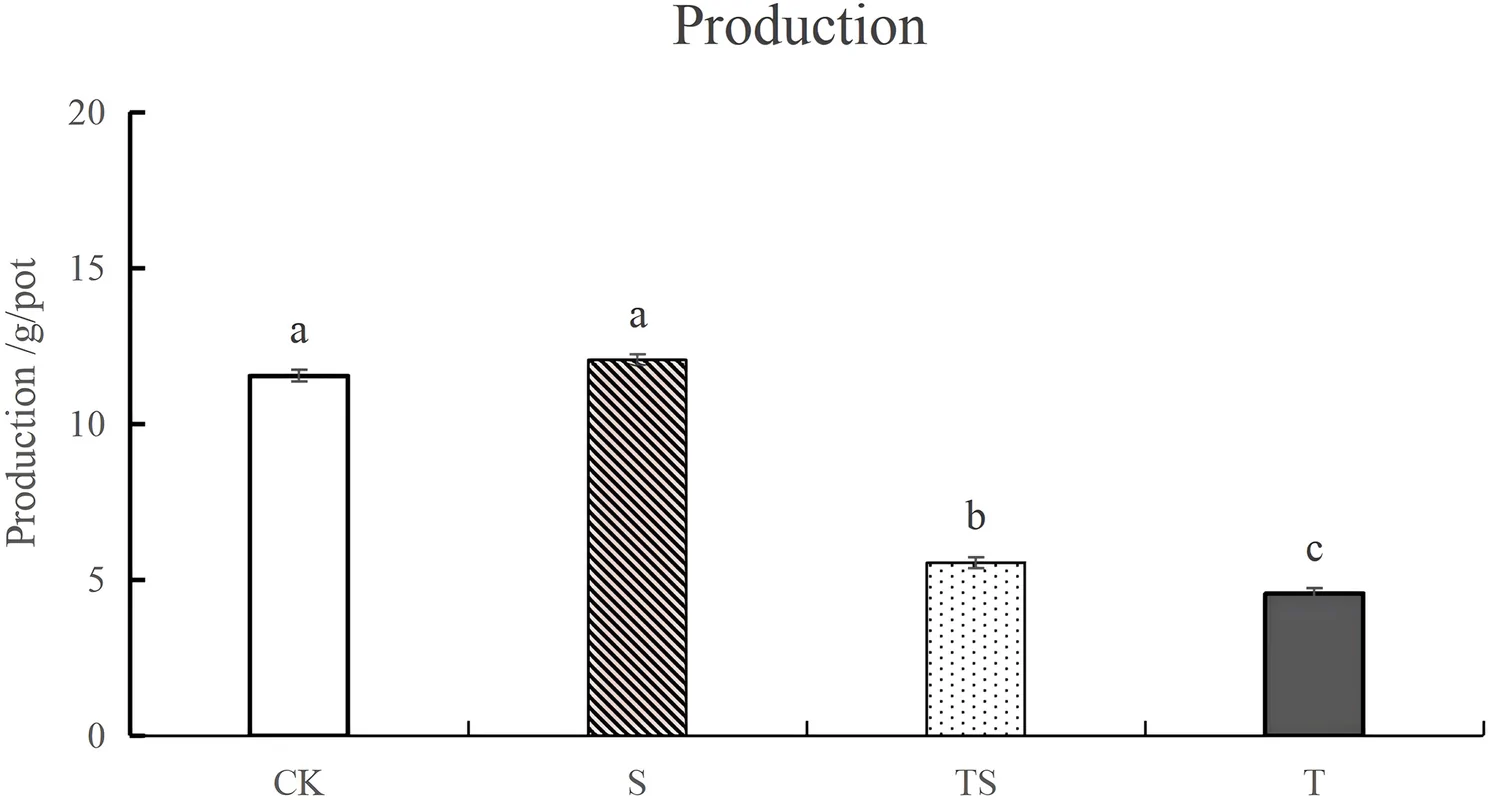
Tuber fresh yield per pot of potato under alkaline-salt stress with or without exogenous Spd application. Data are means ± SD (*n* = 10). Different lowercase letters on bars denote significant differences determined by one-way ANOVA followed by LSD multiple comparison tests at *P* < 0.05. CK, distilled water control; S, foliar application of 1 mmol·L^− 1^ Spd under normal conditions; TS, combined treatment of 300 mmol·L^− 1^ NaHCO_3_ and 1 mmol·L^− 1^ Spd; T, single alkaline-salt stress of 300 mmol·L^− 1^ NaHCO_3_

Under normal growth conditions, potato plants treated with exogenous Spd (S) showed no obvious phenotypic differences from the control (CK), with normal leaf color and robust growth. Under alkaline-salt stress conditions (T), the potato plants exhibited clear stress symptoms, including partial leaf chlorosis (yellowing) and wilting. Plant growth was significantly inhibited, and tuber yield was markedly reduced. Exogenous Spd treatment (TS) effectively alleviated the degree of leaf chlorosis and wilting caused by alkaline-salt stress. The overall growth status of the plants was notably better than that of the T group, and the loss of tuber yield was significantly compensated (Figs. 1 and 2). The results indicate that exogenous Spd can effectively alleviate the inhibition of potato plant growth caused by alkaline-salt stress, which is consistent with previous findings in grapevine [21] and tomato [26].

### Effects of exogenous Spd on MDA, proline, and soluble sugar contents under alkaline-salt stress

**Fig. 3:**
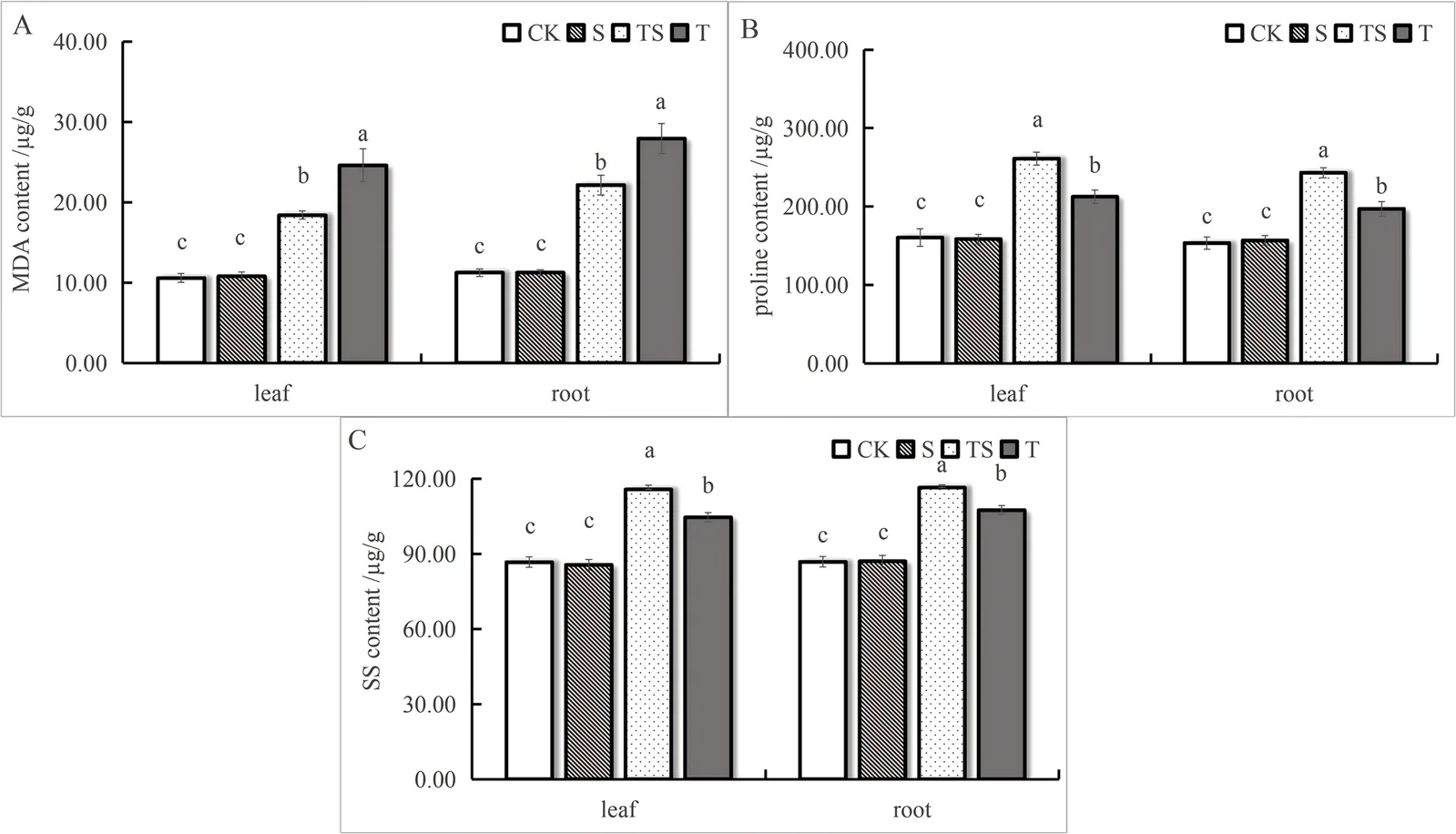
Effects of exogenous Spd on MDA, proline, and soluble sugar contents in potato leaves and roots under alkaline-salt stress. **A** MDA content; (**B**) Proline content; (**C**) Soluble sugar content. Leaf and root tissues were quantified separately. Data are means ± SD (*n* = 3). Different lowercase letters on bars denote significant differences determined by one-way ANOVA followed by LSD multiple comparison tests at *P* < 0.05. CK, distilled water control; S, foliar application of 1 mmol·L^− 1^ Spd under normal conditions; TS, combined treatment of 300 mmol·L^− 1^ NaHCO_3_ and 1 mmol·L^− 1^ Spd; T, single alkaline-salt stress of 300 mmol·L^− 1^ NaHCO_3_

Malondialdehyde (MDA) is a major product of membrane lipid peroxidation, and its content serves as an important indicator of oxidative damage to cell membranes [27]. As shown in Fig. 3A, alkaline-salt stress (T) significantly increased the MDA content in both leaves and roots of potato, whereas exogenous Spd treatment (TS) markedly reduced MDA accumulation. Compared with the T group, the MDA content in the leaf of the TS group decreased by 25.19%, and root MDA content decreased by 20.69%, indicating that exogenous Spd can effectively mitigate membrane lipid peroxidation damage under alkaline-salt stress.

Proline and soluble sugars are important osmotic adjustment substances in plants under stress conditions, maintaining cellular osmotic balance and membrane structural stability [28, 29]. As shown in Fig. 3B, C, alkaline-salt stress (T) induced a significant accumulation of proline and soluble sugars. Following exogenous Spd application (TS), the contents of both osmotic adjustment substances further increased significantly: compared with the T group, proline content in leaves and roots increased by 22.91% and 23.37%, respectively, and soluble sugar content increased by 10.11% and 8.34%, respectively.

### Effects of exogenous Spd on antioxidant enzyme activities under alkaline-salt stress

**Fig. 4:**
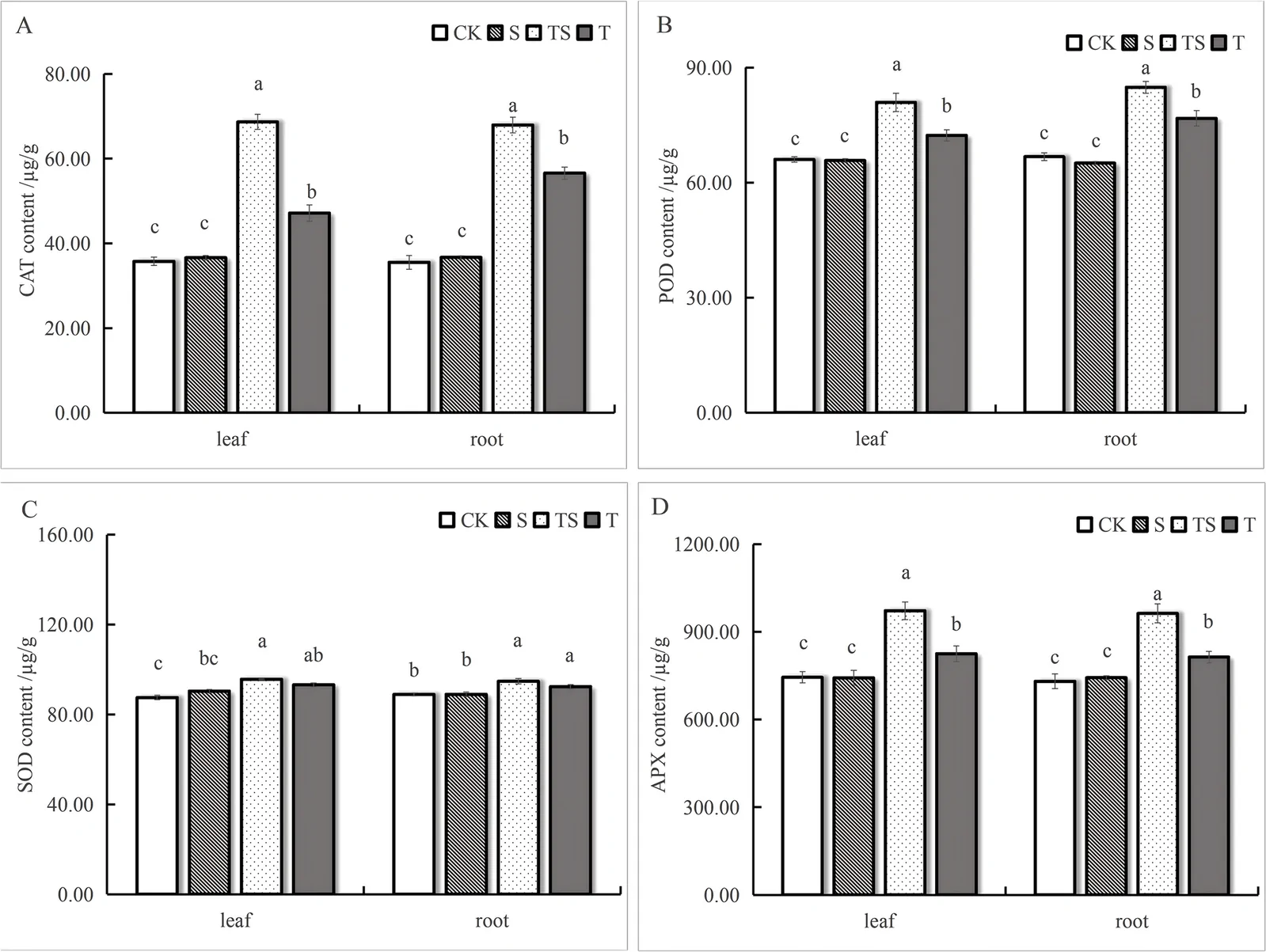
Effects of exogenous Spd on antioxidant enzyme activities in potato leaves and roots under alkaline-salt stress. **A** CAT activity; (**B**) POD activity; (**C**) SOD activity; (**D**) APX activity. Data are means ± SD (*n* = 3). Different lowercase letters on bars denote significant differences determined by one-way ANOVA followed by LSD multiple comparison tests at *P* < 0.05. CK, distilled water control; S, foliar application of 1 mmol·L^− 1^ Spd under normal conditions; TS, combined treatment of 300 mmol·L^− 1^ NaHCO_3_ and 1 mmol·L^− 1^ Spd; T, single alkaline-salt stress of 300 mmol·L^− 1^ NaHCO_3_

The antioxidant enzyme system is a key mechanism for scavenging ROS and defending against oxidative stress in plants [30]. As shown in Fig. 4, alkaline-salt stress (T) activated the antioxidant enzyme system of potato to some extent, whereas exogenous Spd treatment (TS) significantly enhanced the activities of several antioxidant enzymes. Compared with the T group, catalase (CAT) activity in the TS group significantly increased by 45.66% in leaves and 20.05% in roots; peroxidase (POD) activity significantly increased by 11.90% and 10.57%, respectively; and ascorbate peroxidase (APX) activity significantly increased by 17.85% and 18.35%, respectively. However, superoxide dismutase (SOD) activity did not change significantly after exogenous Spd treatment (Fig. 4).

### Transcriptome sequencing and analysis of differentially expressed genes

To comprehensively elucidate the molecular mechanisms by which exogenous Spd enhances alkaline-salt stress tolerance in potato at the transcriptional level, transcriptome sequencing was performed on leaf and root samples from the four treatment groups. A total of 147.51 Gb of clean data was obtained, with the Q30 base percentage of each sample no less than 94.89% (Table S1), and mapping rates ranging from 74.41% to 87.85%.

**Fig. 5:**
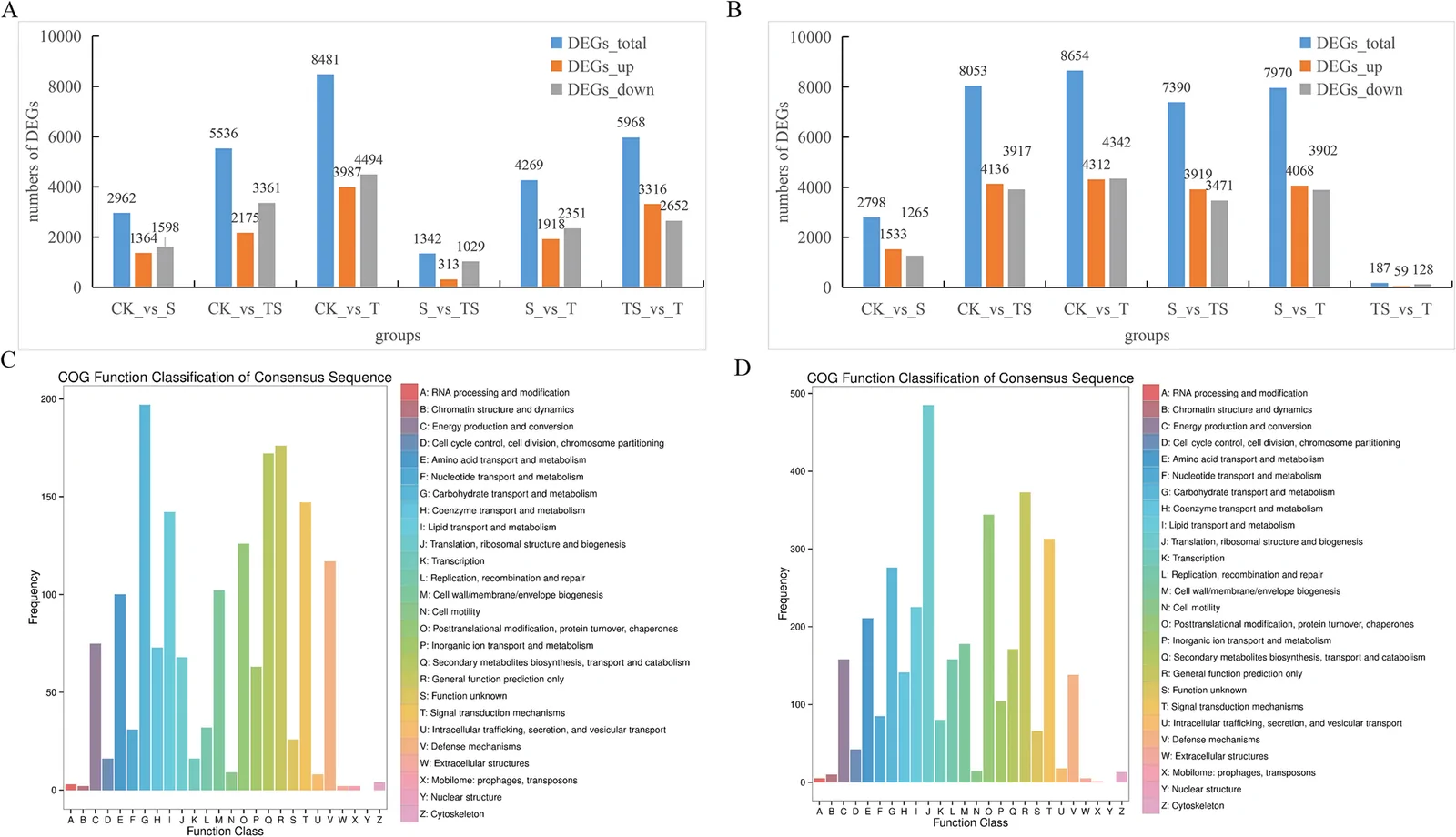
DEG identification and COG functional classification of leaf and root transcriptomes under alkaline-salt stress. **A**, **B** Numbers of upregulated and downregulated DEGs in leaves and roots (TS vs. T); (**C**,**D**) COG functional category distribution of DEGs in leaf and root tissues. CK, distilled water control; S, foliar application of 1 mmol·L^− 1^ Spd under normal conditions; TS, combined treatment of 300 mmol·L^− 1^ NaHCO_3_ and 1 mmol·L^− 1^ Spd; T, single alkaline-salt stress of 300 mmol·L^− 1^ NaHCO_3_

Analysis of differentially expressed genes (DEGs) revealed that in the TS vs. T comparison, 5,968 DEGs were identified in leaves (3,316 upregulated, 2,652 downregulated), while only 187 DEGs were identified in roots (59 upregulated, 128 downregulated) (Fig. 5A, B). The number of DEGs in roots was significantly lower than in leaves. By comparing the control with the alkaline-salt stress condition, 8,481 and 8,654 DEGs were identified in leaves and roots, respectively (leaves: 3,987 upregulated, 4,494 downregulated; roots: 4,312 upregulated, 4,342 downregulated). By comparing Spd treatment with alkaline-salt stress treatment, 5,968 and 187 DEGs were identified in leaves and roots, respectively (leaves: 3,316 upregulated, 2,652 downregulated; roots: 59 upregulated, 128 downregulated). COG functional classification revealed distinct tissue-specific enrichment patterns among DEGs. In leaves, the predominant categories were carbohydrate transport and metabolism (11.53%), secondary metabolite biosynthesis, transport and catabolism (10.06%), signal transduction mechanisms (8.6%), and lipid transport and metabolism (8.31%) (Fig. 5C). In roots, DEGs were mainly enriched in translation, ribosomal structure and biogenesis (13.42%), signal transduction mechanisms (8.66%), posttranslational modification, protein turnover, and chaperones (9.52%), and amino acid transport and metabolism (5.84%) (Fig. 5D). These results indicate that while both tissues share enrichment in signal transduction and carbohydrate metabolism, leaf responses are additionally characterized by pronounced secondary metabolic reprogramming, whereas root responses rely more heavily on protein metabolism machinery.

### KEGG pathway enrichment analysis of DEGs under alkaline-salt stress conditions

**Fig. 6:**
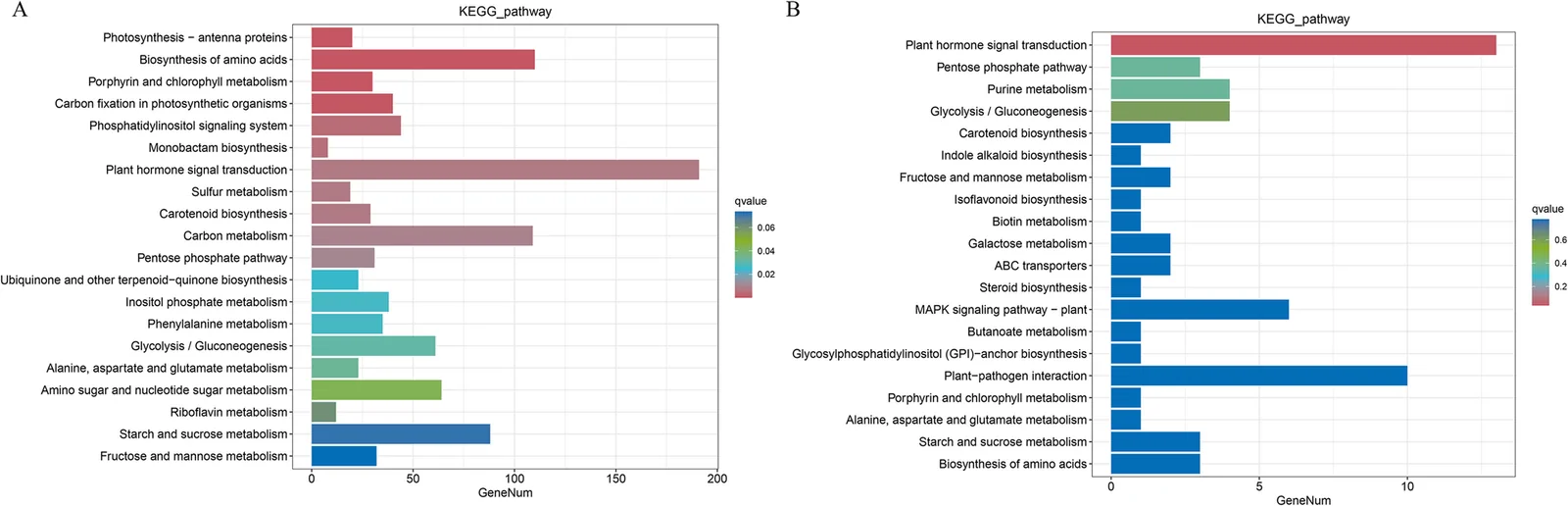
Top enriched KEGG pathways of DEGs derived from the TS vs. T comparison. **A** KEGG enrichment results for leaf DEGs; (**B**) KEGG enrichment results for root DEGs. The x-axis represents the number of enriched genes within each pathway term

KEGG pathway enrichment analysis showed that in leaf tissue, DEGs were mainly enriched in plant hormone signal transduction, carbon metabolism, photosynthesis, and amino acid biosynthesis pathways. Among them, plant hormone signal transduction had the highest number of enriched genes and was the most significantly enriched pathway, followed by carbon metabolism. Concurrently, photosynthesis-related pathways, sugar metabolism pathways, and amino acid metabolism pathways were also significantly enriched, indicating that exogenous Spd may mediate leaf responses to alkaline-salt stress by regulating these pathways (Fig. 6A).

In root tissue, DEGs were mainly enriched in ribosome, spliceosome, proteasome, oxidative phosphorylation, and amino acid biosynthesis pathways. The ribosome pathway had the highest number of enriched genes and was the most predominantly enriched pathway, followed by the spliceosome pathway. In addition, pathways such as oxidative phosphorylation, RNA transport, carbon metabolism, and amino acid biosynthesis were also significantly enriched, indicating that roots mainly respond to stress under alkaline-salt stress and exogenous Spd regulation by activating protein synthesis and degradation, energy metabolism, and basal metabolic pathways (Fig. 6B).

### Validation of transcriptome data reliability by qRT-PCR

**Fig. 7:**
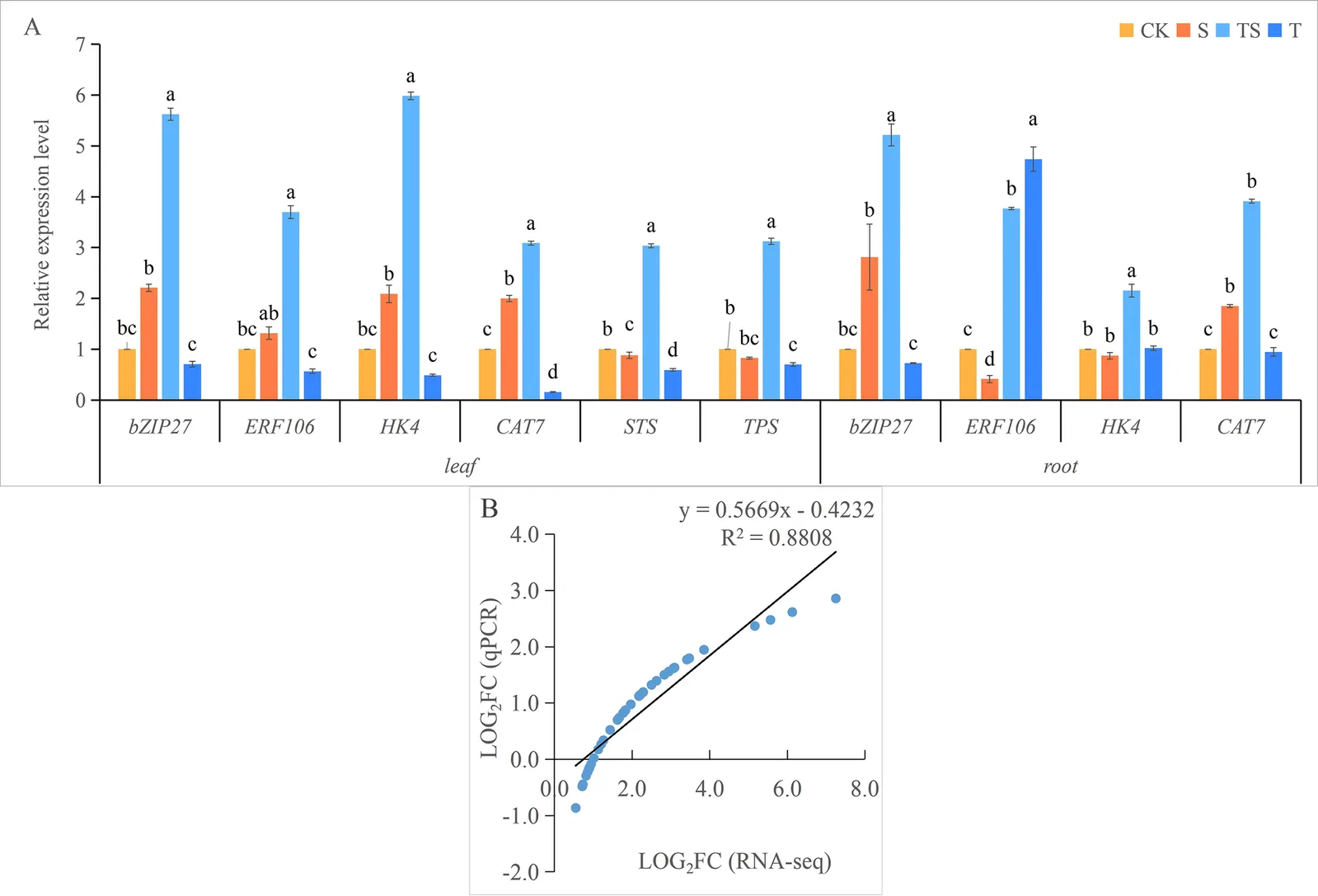
qRT-PCR validation of transcriptome sequencing data. **A** Relative expression levels of six representative hub genes in potato leaves and roots across four treatments. **B** Correlation analysis of log_2_ expression values between RNA-seq and qRT-PCR results. Data are means ± SD (*n* = 3). Different lowercase letters on bars denote significant differences determined by one-way ANOVA followed by LSD multiple comparison tests at *P* < 0.05. CK, distilled water control; S, foliar application of 1 mmol·L^− 1^ Spd under normal conditions; TS, combined treatment of 300 mmol·L^− 1^ NaHCO_3_ and 1 mmol·L^− 1^ Spd; T, single alkaline-salt stress of 300 mmol·L^− 1^ NaHCO_3_

Six differentially expressed genes were selected for qRT-PCR validation (Fig. 7A). The results showed a high degree of consistency between qRT-PCR and RNA-seq data (*R²= 0.8808*) (Fig. 7B), confirming the reliability of the transcriptome sequencing data.

### Combined analysis of WGCNA core modules and DEGs in leaves and roots

**Fig. 8:**
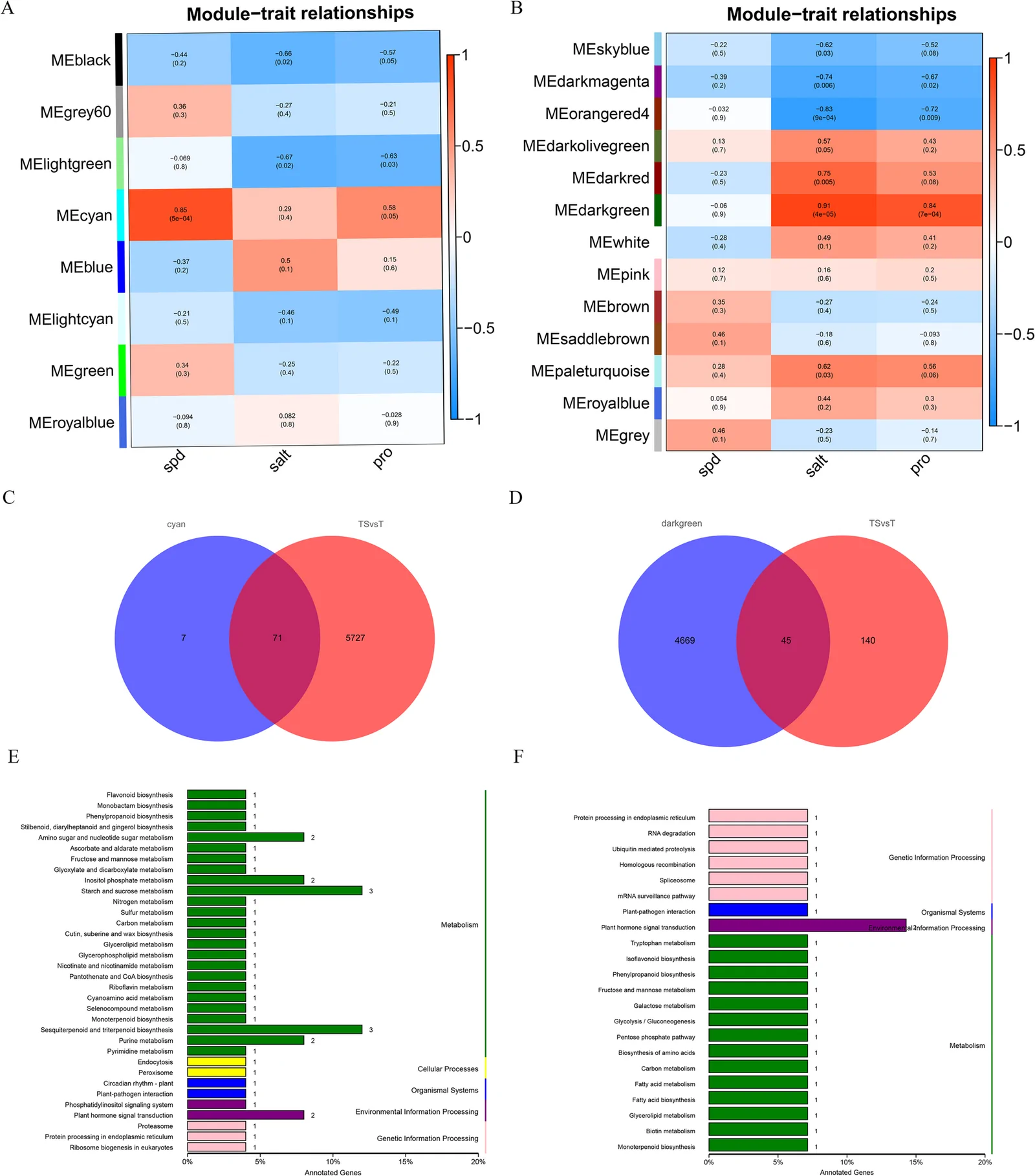
WGCNA and functional enrichment of core Spd-responsive modules. **A**, **B** Correlation heatmaps between module eigengenes and phenotypic traits for leaf MEcyan and root MEdarkgreen modules. Each cell shows the correlation coefficient and corresponding P-value. **C**, **D** Venn diagrams of overlapping genes between core module genes and TS vs. T DEGs in leaves and roots. **E**, **F** KEGG pathway enrichment of overlapping core candidate genes

The results of weighted gene co-expression network analysis (WGCNA) exhibited clear tissue specificity in leaves and roots. In leaf tissue, the MEcyan module showed the strongest correlation with exogenous Spd and proline content traits (*r = 0.85, P = 5 × 10*^− 4^) (Fig. 8A), indicating that this module is the core co-expression module responding to exogenous Spd regulation in leaves. In root tissue, the MEdarkgreen module was significantly positively correlated with salt stress and proline content traits (*r = 0.91, P = 4 × 10*^− 5^), indicating that this module is the key module responding to salt stress in roots (Fig. 8B).

To further identify core genes specifically responsive to exogenous Spd regulation under alkaline-salt stress in potato, the core module genes obtained by WGCNA were intersected with the DEGs from the TS vs. T comparison. In leaves, the intersection of MEcyan module genes and DEGs yielded 71 overlapping genes, while in roots, the intersection of MEdarkgreen module genes and DEGs yielded 45 overlapping genes (Fig. 8C, D).

To further dissect the core molecular mechanisms underlying the potato response to exogenous Spd under alkaline-salt stress, KEGG pathway enrichment analysis was performed on the intersected genes between WGCNA core modules and DEGs in both leaves and roots. In leaves, the 71 core intersecting genes were predominantly enriched in metabolic pathways, including starch and sucrose metabolism, sesquiterpenoid and triterpenoid biosynthesis, inositol phosphate metabolism, and amino sugar metabolism. Concurrently, the plant hormone signal transduction pathway was also significantly enriched, suggesting that Spd may mediate leaf responses to alkaline-salt stress by coordinately modulating primary metabolism, secondary metabolism, and hormone signaling networks (Fig. 8E). In roots, the 45 core intersecting genes were most significantly enriched in the plant hormone signal transduction pathway, along with genetic information processing pathways (e.g., protein processing in the endoplasmic reticulum, RNA degradation) and primary metabolic pathways (e.g., glycolysis, amino acid biosynthesis) (Fig. 8F). This enrichment pattern indicates that roots respond to alkaline-salt stress primarily through rapid activation of hormone signal transduction, coupled with the coordinated regulation of protein metabolism and basal energy metabolism to initiate stress responses.

Module-trait relationships in leaf (A) and root (B) tissues. Each cell shows the correlation coefficient (top) and p-value (bottom) between the module eigengene and the traits (spd, salt, pro). Venn diagrams showing the overlap between genes in the cyan module (leaf) / darkgreen module (root) and the TS vs. T DEGs. KEGG pathway enrichment analysis of the overlapping genes from leaf (E) and root (F) tissues.

These pathway-level results enabled the screening of core candidate genes responding to Spd under alkaline-salt stress. Representative genes with altered expression patterns are listed in Table 1. The most strongly upregulated gene was *histidine kinase 4* (*StHK4*; Log_2_FC = 3.40), which functions in phytohormone signal perception and transduction. Notably, two transcription factors, *StERF106* (Log_2_FC = 2.23) and *StbZIP27* (Log_2_FC = 2.12), were also significantly induced. In addition, several genes involved in ion homeostasis, terpenoid metabolism, and antioxidant defense were also upregulated, while a small set of genes related to secondary metabolism and redox homeostasis (e.g., *MYB113*, Log_2_FC = -5.43) were downregulated (Table 1). These candidate genes provide valuable targets for further investigation of the molecular mechanisms underlying Spd-enhanced alkaline-salt tolerance in potato.

**Table 1:** Key candidate genes associated with Spd-mediated alkaline salt stress tolerance in potato

Gene ID	Log₂ (fold change)	Expression pattern	Gene name	Functional annotation
*Soltu.DM.04G003490*	3.40	Up-regulated	*Histidine kinase 4*	Mediates perception and transduction of phytohormone signals during stress adaptation
*Soltu.DM.11G006920*	3.16	Up-regulated	*Cationic amino acid transporter 7*	Amino acid transporter involved in the transport of osmoprotectants for osmotic adjustment
*Soltu.DM.09G008230*	2.32	Up-regulated	*Vacuolar cation/proton exchanger *	Vacuolar antiporter that modulates ion homeostasis under salt stress
*Soltu.DM.05G022060*	2.23	Up-regulated	*ERF106*	Ethylene-responsive transcription factor that mediates signal responses to abiotic stress
*Soltu.DM.02G005680*	2.12	Up-regulated	*bZIP transcription factor 27*	A core transcription factor in the ABA signaling pathway, involved in transcriptional regulation under abiotic stress
*Soltu.DM.09G029830*	2.08	Up-regulated	*Sesquiterpene synthase*	Regulates the biosynthesis of defensive secondary metabolites under stress conditions
*Soltu.DM.09G029760*	1.97	Up-regulated	*Terpene synthase*	Essential enzyme of secondary metabolism in response to abiotic stress
*Soltu.DM.06G012170*	1.55	Up-regulated	*Chloroplastic Fe-SOD*	Chloroplastic Fe-superoxide dismutase; scavenges ROS and alleviates oxidative damage under stress
*Soltu.DM.10G020780*	−5.43	Down-regulated	*MYB113*	MYB transcription factor modulating secondary metabolism and stress defense responses
*Soltu.DM.03G006230*	−2.33	Down-regulated	*Inositol monophosphatase 2*	Participates in inositol metabolism and regulates osmotic signaling under stress
*Soltu.DM.05G006570*	−1.97	Down-regulated	*Thioredoxin H2*	Maintains cellular redox homeostasis in response to abiotic stress

## Discussion

Alkaline-salt stress severely disturbs rhizosphere physicochemical properties by elevating soil pH and Na^+^ levels, thereby disrupting plant water balance, ion homeostasis, and nutrient absorption, and ultimately suppressing plant growth and tuber productivity [12, 31]. In this study, alkaline-salt stress induced typical stress phenotypes including leaf chlorosis and growth inhibition, accompanied by significant tuber yield reduction in potato cultivar ‘Huishu No. 5’, Exogenous Spd application effectively alleviated stress-induced physiological damage and yield loss (Fig. 2), which is consistent with previous reports in multiple crop species [21, 24, 32–34]. The treatment concentrations used in this study were determined based on published protocols and preliminary screening, ensuring stable stress phenotypes and no phytotoxicity, which guarantees the reliability of the phenotypic and physiological analyses.

Oxidative damage and osmotic imbalance are two dominant physiological constraints under alkaline-salt stress. Stress-induced ROS overaccumulation triggers plasma membrane lipid peroxidation, and elevated MDA content reflects the degree of membrane oxidative injury [35]. In agreement with previous studies [36, 37], our results showed that Spd reduced MDA accumulation in both leaves and roots, indicating that Spd mitigates membrane peroxidation damage. Additionally, proline and soluble sugars act as vital osmoprotectants for maintaining cellular osmotic stability under salt-related stress [18, 38]. Exogenous Spd further increased the accumulation of these osmolytes under stress (Fig. 3B, C), suggesting that Spd improves osmotic adjustment capacity to counteract alkaline-salt-induced osmotic injury.

Antioxidant enzymes including SOD, POD, CAT and APX constitute the primary enzymatic ROS-scavenging system in plants [39]. Exogenous Spd can further enhance antioxidant enzyme activities under salt stress in cucumber, tomato and other species [37, 40–42]. Spd significantly increased CAT, POD, and APX activities in both potato leaves and roots under alkaline-salt stress (Fig. 4), thereby enhancing plant antioxidant defense capacity.

Transcriptomic analysis revealed pronounced tissue-specific transcriptional reprogramming underlying Spd-mediated stress tolerance. The dramatically higher number of DEGs in leaves (5,968) than in roots (187) indicates that leaves are the primary tissue perceiving Spd signals, while roots exhibit relatively mild transcriptional responses through long-distance signal crosstalk [11]. KEGG enrichment analysis suggested that leaf Spd-responsive genes are mainly involved in hormone signal transduction, photosynthesis, and carbon metabolism, whereas root DEGs are enriched in ribosome biogenesis and energy metabolism pathways, highlighting distinct tissue adaptive strategies under combined Spd and alkaline-salt treatment [43, 44].

Integrated physiological and transcriptomic analysis further revealed divergent antioxidant regulatory patterns between leaves and roots. In leaves, Spd transcriptionally upregulated a series of antioxidant enzyme genes, including CAT, APX, POD, and SOD family members, which directly corresponded to elevated antioxidant enzyme activities (Fig. 4). Notably, the significant induction of thylakoid-localized APX gene indicates that Spd specifically enhances chloroplastic ROS-scavenging ability and protects photosynthetic apparatus under stress [45]. In contrast, root antioxidant responses exhibited obvious transcript-physiology uncoupling. Spd barely induced the expression of core antioxidant genes in roots, suggesting that root antioxidant improvement is largely independent of transcriptional regulation. This tissue-divergent response is consistent with previous salt stress studies in *Brassica napus* [46]. Root antioxidant homeostasis is likely modulated mainly via post-translational modification and non-enzymatic antioxidant systems, which is consistent with the proposed Spd regulatory mechanism in root stress adaptation [47, 48].

Exogenous Spd also improved alkaline-salt tolerance by reprogramming genes related to polyamine catabolism, osmolyte transport, and ion homeostasis. Spd-induced polyamine oxidase upregulation facilitates proline precursor Δ^1^-pyrroline accumulation and promotes proline biosynthesis [49]. Meanwhile, Spd-induced *CAT7* and *CAX3* expression contributes to osmolyte redistribution and vacuolar Na sequestration, alleviating cytoplasmic ion toxicity and maintaining osmotic balance [50, 51]. Combined with the increased proline and soluble sugar contents in Spd-treated seedlings, these results indicate that Spd strengthens osmotic adjustment via multi-pathway transcriptional regulation.

WGCNA analysis identified distinct tissue-specific Spd-responsive modules, with the leaf MEcyan module and root MEdarkgreen module tightly correlated with Spd response and stress adaptation, respectively. The intersection of hub genes and DEGs yielded 71 and 45 core candidate genes in leaves and roots, among which *StHK4*, *StbZIP27*, and *StERF106* were identified as candidate central regulatory nodes for Spd-mediated alkaline-salt tolerance. Similar transcriptome-WGCNA strategies have been widely applied for salt-response gene screening, supporting the rationality of our screening strategy [52].

*StHK4* exhibited the highest upregulation (Log_2_FC = 3.40) under Spd treatment, and may function as a potential upstream sensor linking Spd signal perception to downstream stress regulation. It is orthologous to Arabidopsis cytokinin receptor *AHK4/CRE1*, which mediates two-component phosphorelay signal transduction in stress responses [53, 54]. Cytokinin signaling antagonizes ABA-dependent salt stress responses and modulates proline accumulation and root growth under salinity [55]. The strong Spd-induced upregulation of *StHK4*, together with its co-expression with proline content in our WGCNA analysis, suggests that *StHK4* may function as a potential upstream regulatory factor that links Spd perception to cytokinin-mediated regulation of osmotic adjustment, possibly through crosstalk with ABA-dependent stress pathways.

*StbZIP27*, a member of the bZIP transcription factor family, regulates multiple plant abiotic stress responses [56]. In Arabidopsis, direct experimental evidence has demonstrated that bZIP transcription factors regulate proline metabolism under osmotic stress by binding to the promoters of proline metabolic genes such as *ProDH* [57, 58]. Meanwhile, bZIP members from diverse plant species have been shown to enhance ROS-scavenging capacity under salt stress [23, 49]. In potato, *StbZIP* transcription factors enhance saline-alkali tolerance by promoting antioxidant enzyme activities and proline accumulation [23]. In rice, Spd enhances the RNA-binding capacity of bZIP transcription factor *OsbZIP73* under salt stress [48], which provides a direct link between polyamine metabolism and bZIP-dependent stress regulatory pathway. Our finding that Spd up-regulates *StbZIP27* suggests it transcriptionally activates downstream osmotic adjustment and ROS-scavenging genes to contribute to improved alkaline-salt tolerance in potato.

*StERF106* belongs to the *AP2/ERF* ethylene-responsive transcription factor superfamily. In Solanaceous crops, ERF transcription factors have been demonstrated to improve salt tolerance by enhancing ROS detoxification, as evidenced by overexpression of *SlERF84* in tomato and *StERF94* in potato, both of which elevated antioxidant enzyme activities and reduced ROS accumulation under salt stress [59, 60]. Furthermore, in rice, *OsERF106MZ* has been directly implicated in the regulation of ROS levels and CAT activity under salinity stress [61], providing direct evidence that *ERF106*-class genes participate in ROS homeostasis. Combined with physiological data showing elevated CAT, POD and APX activities upon Spd application, it is proposed that *StERF106* may serve as a potential mediator coordinating ethylene signaling and Spd-induced antioxidant defense responses to maintain ROS homeostasis under alkaline-salt stress.

The foliar Spd protocol in this study was established under controlled greenhouse conditions, and its feasibility for field-scale application warrants further investigation. Nevertheless, the effective Spd concentration is low, the compound is commercially available, and alternative low-cost delivery methods, such as seed priming, have proven effective in field crops. More importantly, the candidate genes identified in this study provide valuable targets for potato breeding programs, offering a sustainable long-term strategy for enhancing alkaline-salt tolerance. In addition, the precise biological functions of *StHK4*, *StbZIP27*, and *StERF106* require further verification through gene overexpression and knockout assays in future functional studies.

## Conclusion

**Fig. 9:**
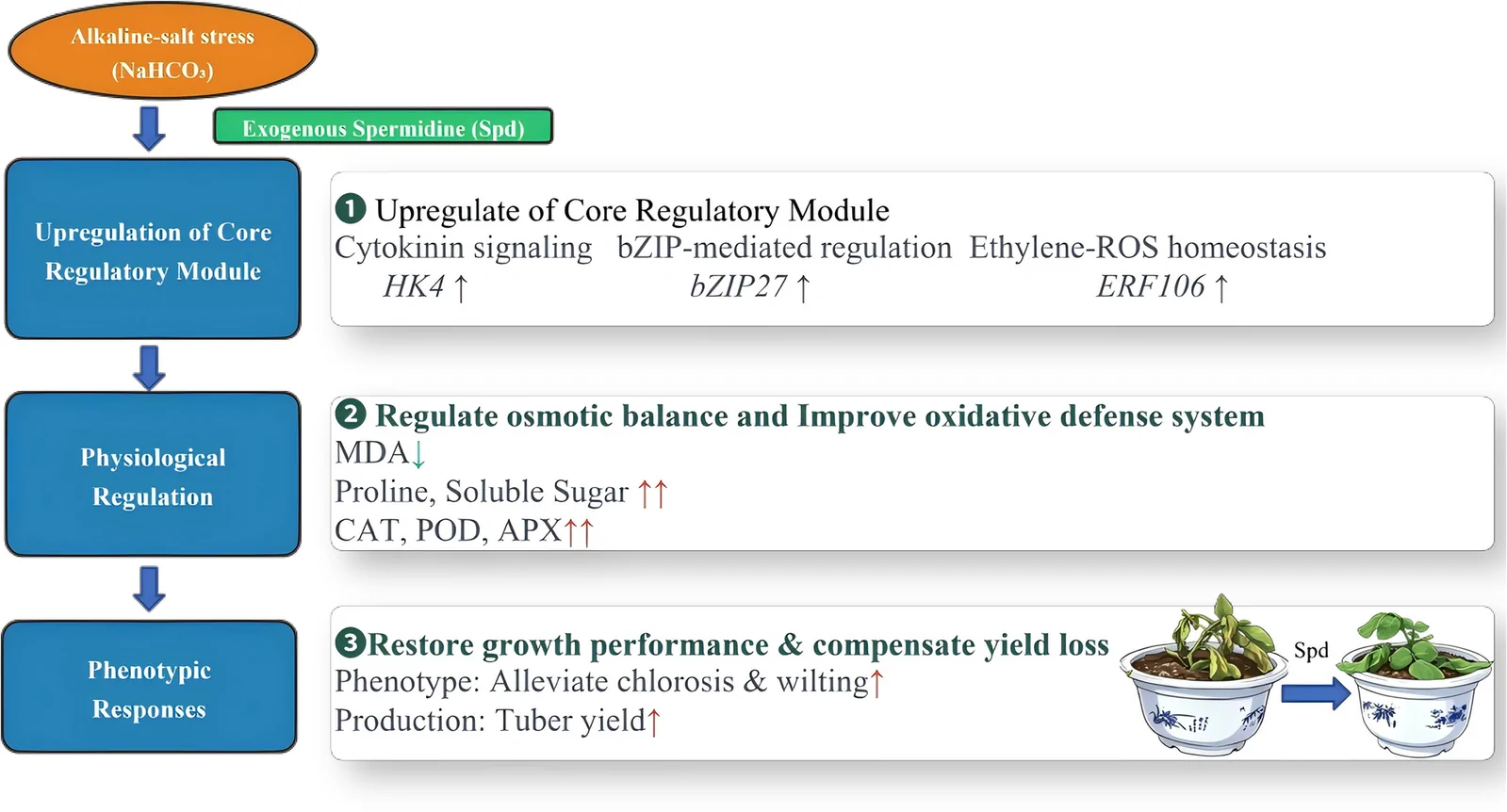
Schematic regulatory model of exogenous spermidine (Spd) conferring alkaline-salt stress tolerance in potato

This study identifies three candidate hub genes (*StHK4*, *StbZIP27* and *StERF106*) potentially participating in Spd-mediated alkaline-salt tolerance in potato based on transcriptome and WGCNA analysis. Exogenous Spd improves cell membrane stability, osmotic adjustment capacity and ROS-scavenging activity through tissue-divergent transcriptional and physiological pathways, thereby alleviating leaf oxidative injury and restoring tuber productivity under alkaline-salt stress (Fig. 9). This study advances the mechanistic understanding of polyamine-regulated stress tolerance and supplies candidate genes for follow-up functional assays and salt-tolerant potato breeding.

## Supplementary Information

Supplementary Material 1.

## Data Availability

The raw transcriptomic sequence data generated in this study have been deposited in the NCBI Sequence Read Archive (SRA) under BioProject accession number PRJNA1471356. The BioProject is accessible at the following URL: https://www.ncbi.nlm.nih.gov/bioproject/PRJNA1471356.
