# Supplementary material for: Exogenous spermidine (Spd) improves the tolerance to alkaline-salt stress in potato
Source: BMC Plant Biol. 2026 Aug 4;26:1568. doi: 10.1186/s12870-026-09656-7 (PMC13560374; doi:10.1186/s12870-026-09656-7)
Supplement: Supplementary file 1 [file 12870_2026_9656_MOESM1_ESM.docx]

Supplementary Table S1. Summary of RNA-seq data quality control statistics

| Samples | Clean reads | Clean bases | GC Content | %≥Q30 |
| --- | --- | --- | --- | --- |
| CK-R-1 | 20,727,630 | 6,203,117,606 | 41.78% | 96.27% |
| CK-R-2 | 19,630,633 | 5,875,446,536 | 42.33% | 96.16% |
| CK-R-3 | 20,337,840 | 6,091,814,266 | 41.53% | 96.37% |
| CK-S-1 | 20,547,218 | 6,119,426,831 | 42.51% | 96.34% |
| CK-S-2 | 21,335,923 | 6,345,756,783 | 42.97% | 96.16% |
| CK-S-3 | 20,620,252 | 6,116,702,989 | 42.91% | 96.39% |
| S-R-1 | 20,255,006 | 6,062,798,295 | 41.57% | 95.85% |
| S-R-2 | 19,945,587 | 5,966,346,626 | 41.64% | 94.89% |
| S-R-3 | 20,878,639 | 6,244,446,782 | 41.58% | 96.64% |
| S-S-1 | 20,614,749 | 6,124,062,359 | 42.57% | 96.37% |
| S-S-2 | 19,930,817 | 5,968,884,169 | 42.93% | 96.55% |
| S-S-3 | 20,909,647 | 6,211,664,585 | 42.57% | 96.30% |
| TS-R-1 | 20,320,572 | 6,062,015,234 | 42.57% | 96.05% |
| TS-R-2 | 21,215,433 | 6,334,426,740 | 42.48% | 95.95% |
| TS-R-3 | 20,784,208 | 6,208,240,116 | 41.87% | 95.96% |
| TS-S-1 | 20,291,074 | 6,043,478,028 | 42.79% | 96.35% |
| TS-S-2 | 20,848,960 | 6,196,477,079 | 42.74% | 96.35% |
| TS-S-3 | 20,566,113 | 6,097,050,588 | 42.81% | 96.36% |
| T-R-1 | 21,471,840 | 6,416,219,620 | 42.60% | 96.15% |
| T-R-2 | 21,588,441 | 6,465,791,029 | 43.24% | 96.52% |
| T-R-3 | 19,821,121 | 5,921,316,701 | 42.40% | 96.01% |
| T-S-1 | 21,017,682 | 6,249,674,867 | 42.31% | 96.11% |
| T-S-2 | 20,879,891 | 6,218,143,571 | 42.39% | 96.40% |
| T-S-3 | 20,166,718 | 5,969,786,898 | 42.36% | 96.26% |

Abbreviations: CK, control group; S, alkaline salt stress group; TS, alkaline salt stress + Spd treatment group; T, Spd treatment group; R, root; S, leaf.

Supplementary Table S2. Primer sequences used for qRT‑PCR validation

| Gene ID | Forward primer | Reverse primer |  |  |
| --- | --- | --- | --- | --- |
| Actin | ATTGGAAACGGATATGCTCCA | TCCTTACCTGAACGCCTGTCA |  |  |
| Soltu.DM.02G005680 | AAAGAACCGTGAGTCCGCTG | TAACTGTTGCTGCTGCCTCTT |  |  |
| Soltu.DM.05G022060 | TCAGTCCAGTTTCCGACAGC | GGATCTCTTATCTCCGCCGC |  |  |
| Soltu.DM.04G003490 | CAAAAAGTTTGGGGCCGAGG | GACGTGTAGCCTCAAACCCA |  |  |
| Soltu.DM.11G006920 | CACTGAGTTCGCCGTCGATA | TCAGATTCGCACCGGTCAAA |  |  |
| Soltu.DM.09G029830 | TCTGGGGTGTATTTTGGGCC | ATCTCTCAACTGCGTCGGTG |  |  |
| Soltu.DM.09G029760 | CAGGGGTGTATTTTGGGCCA | AACTGCATCGGTGTAAGGCA |  |  |
